# Chronic kidney disease and small renal tumors: What urologists should know?

**DOI:** 10.4103/0970-1591.57913

**Published:** 2009

**Authors:** K. Muruganandham, Anil Mandhani

**Affiliations:** Department of Urology, Sanjay Gandhi Post Graduate Institute of Medical Sciences, Lucknow, UP, India

**Keywords:** Chronic kidney disease, partial nephrectomy, small renal tumors

## Abstract

**Objective:**

To review the evidence in literature regarding the occurrence of Chronic Kidney Disease (CKD) after the treatment of small renal tumors with either radical nephrectomy (RN) or partial nephrectomy (PN).

**Materials and Methods:**

Current literature reviewed using Mediline search regarding renal functional outcomes following surgical treatment of small renal tumours.

**Results:**

Studies have clearly shown that RN leads to CKD more often than PN and RN remains an independent risk factor for patients developing new onset renal insufficiency. There is independent, graded association between a reduced estimated GFR and the risk of death, cardiovascular events, and hospitalization. PN achieves a better Health Related Quality Of Life due to better preservation of renal function. Radical nephrectomy is significantly associated with death from any cause compared with partial nephrectomy.

**Conclusion:**

Removal of entire kidney is definitely an over-treatment for small renal tumors and PN should be the standard of care for these small renal tumors even in the setting of a normal contralateral kidney.

## INTRODUCTION

Small renal tumors (SRT) that are 4 cm or smaller now account for the largest proportion of newly diagnosed renal masses.[1] Despite the fact that a partial nephrectomy (PN) is not the most common approach to treat such tumors, contemporary data indicate that only 20% of renal tumors 2 to 4 cm are treated with PN in the United States and only 4% of all nephrectomies performed in England use a nephron sparing approach.[2] In spite of the downward size and stage migration of renal tumors, there is no parallel reduction in mortality. A possible reason for this could be a non oncological outcome following the removal of nephron in excess and the resulting development of chronic kidney disease (CKD). In this article, we will review the evidence in literature regarding the occurrence of CKD after the treatment of small renal tumors with a radical nephrectomy (RN).

## EVIDENCE

### Does a radical nephrectomy lead to chronic kidney disease?

In a retrospective study from the Mayo Clinic with 10-year follow-up data that compared the outcome of RNs (n=1492) and PNs (n=189) for unilateral single tumors with normal contralateral kidneys, patients who underwent a RN had a significantly higher risk for proteinuria and CKD i.e., creatinine of more than 2.0 mg/dL (22.4% and 11.6%; p = 0.01).[3] A similar study by McKiernan, *et al.* from Memorial Sloan-Kettering Cancer Center also showed that when controlled for preoperative risk factors for renal insufficiency i.e., hypertension, diabetes etc., patients undergoing a RN are at a greater risk of CKD than a similar cohort of patients undergoing a PN.[4] These studies have clearly shown that a RN leads to CKD more often than a PN despite serum creatinine used as a measure of renal function. The actual incidence would have been more than what was reported as clinically relevant reduction in kidney function can occur in patients even with serum creatinine concentrations of lower than 2 mg%.

Current guidelines define a glomerular filtration rate (GFR) of lower than 60 mL/min per 1·73 m^2^ as Stage 3 CKD.[5] Huang, *et al.* conducted a retrospective cohort study of 662 patients with a normal concentration of serum creatinine who had elective partial or radical nephrectomy for a solitary, renal cortical tumor (≤4 cm). They reported that a RN was significantly associated with new onset GFR less than 45 ml per minute compared with a PN (HR 11.8). The estimated 5-year probability of a postoperative GFR less than 45 ml per minute was 7% *vs.* 43% in patients who underwent a PN *vs.* a RN. A multivariable analysis showed that a RN remained an independent risk factor for patients developing new onset renal insufficiency.[6] In another study by Lucas, *et al.* it has been shown that preoperatively Stage 3 CKD was identified in 26.7% of patients undergoing treatment for small renal tumors. Following intervention, the 3-year freedom from a GFR decrease of below 60 ml per minute per 1.73 m^2^ for radio frequency ablation, PN and RN was 95.2%, 70.7%, and 39.9%, respectively.[7]

### Does renal insufficiency increase the risk of death?

In a large, community-based population study of more than one million patients Go, *et al.* found an independent, graded association between a reduced estimated GFR and the risk of death, cardiovascular events, and hospitalization. Patinets with Stage 3 CKD, which means a GFR of <60 ml/m2, had a 10% increased risk of hospitalization, a 40% increased risk for cardiovascular events, and a 20% increased risk of death as compared with those who had a GFR of >60 ml/m^2^.[8] The association of kidney function with incident hip fracture was examined among a community-based cohort of older individuals. Kidney dysfunction, as assessed by cystatin C, was associated with an increased risk for hip fracture even in patients who have not progressed to dialysis.[9] In a combined analysis of 39 studies that compared RN and PN, Lesage, *et al.* found that a partial nephrectomy achieves a better Health Related Quality Of Life (HRQOL) due to better preservation of renal function and overall quality of life.[10]

### Does a partial nephrectomy improve overall survival?

In a recent study of 648 patients (RN in 290 patients and PN in 358 patients), Thompson, *et al.* compared overall survival in patients with sporadic, unilateral, solitary, and localized renal masses of 4 cm or less that have been treated by RN or PN. In patients younger than 65 years old, a RN was significantly associated with death from any cause compared with a PN (RR 2.16; p = 0.02). The increased risk of death persisted after adjusting for year of surgery, preoperative creatinine, Charlson-Romano index, symptoms at presentation, diabetes at presentation, and histology.[11] The finding of increased risk of death after a RN when compared with a PN was also shown by analysis of 2991 patients older than 66 years of age (RN in 2,547 patients and PN in 556 patients) from the SEER database. When adjusting for preoperative demographic and comorbid variables, RN was associated with an increased risk of overall mortality (HR 1.38: p = 0.01) and a 1.4 times greater number of cardiovascular events after surgery (p = 0.05).[12] These two recent articles demonstrate the direct evidence that RNs result in a higher overall mortality in both younger and older patients.

These data indicate that many patients are at risk for CKD after having a RN as treatment for small, renal cortical tumors. Therefore, these findings should have important implications on the contemporary management of these tumors. Preoperative GFR estimates should be an integral part of the decision algorithm during the planning of the surgical strategy for patients with renal cortical tumors, irrespective of tumor size.

Based on the increasing number of studies showing long-term deleterious effects of CKD, urologists should be aware of the fact that normal serum creatinine may not necessarily mean normal GFR for the age and blanket treatment of small, renal cortical tumors as a RN could have a substantial effect on the quality and length of the survival of these patients. Collectively, these observations show that the removal of the entire kidney, even if performed laparoscopically, is definitely an overtreatment for small renal tumors and a PN should be the standard of care for these small renal tumors even in the setting of a normal contralateral kidney.

## References

[1] Nguyen MM, Gill IS, Ellison LM (2006). The evolving presentation of renal carcinoma in the United States: trends from the Surveillance, Epidemiology, and End Results program. J Urol.

[2] Miller DC, Hollingsworth JM, Hafez KS, Daignault S, Hollenbeck BK (2006). Partial nephrectomy for small renal masses: an emerging quality of care concern?. J Urol.

[3] Lau WK, Blute ML, Weaver AL, Torres VE, Zincke H (2000). Matched comparison of radical nephrectomy vs nephron- sparing surgery in patients with unilateral renal cell carcinoma and a normal contralateral kidney. Mayo Clin Proc.

[4] McKiernan J, Simmons R, Katz J, Russo P (2002). Natural history of chronic renal insufficiency after partial and radical nephrectomy. Urology.

[5] Levey AS, Coresh J, Balk E, Kausz AT, Levin A, Steffes MW (2003). National Kidney Foundation practice guidelines for chronic kidney disease: evaluation, classification, and stratification. Ann Intern Med.

[6] Huang WC, Levey AS, Serio AM, Snyder M, Vickers AJ, Raj GV (2006). Chronic kidney disease after nephrectomy in patients with renal cortical tumors: a retrospective cohort study. Lancet Oncol.

[7] Lucas SM, Stern JM, Adibi M, Zeltser IS, Cadeddu JA, Raj GV (2008). Renal function outcomes in patients treated for renal masses smaller than 4 cm by ablative and extirpative techniques. J Urol.

[8] Go AS, Chertow GM, Fan D, McCulloch CE, Hsu CY (2004). Chronic kidney disease and the risks of death, cardiovascular events, and hospitalization. N Eng J Med.

[9] Fried LF, Biggs ML, Shlipak MG, Seliger S, Kestenbaum B, Stehman-Breen C (2007). Association of kidney function with incident hip fracture in older adults. J Am Soc Nephrol.

[10] Lesage K, Joniau S, Fransis K, Van Poppel H (2007). Comparison between open partial and radical nephrectomy for renal tumors: perioperative outcome and health-related quality of life. Eur Urol.

[11] Thompson RH, Boorjian SA, Lohse CM, Leibovich BC, Kwon ED, Cheville JC (2008). Radical nephrectomy for pT1a renal masses may be associated with decreased overall survival compared with partial nephrectomy. J Urol.

[12] Huang WC, Elkin EB, Levey AS, Jang TL, Russo P (2009). Partial nephrectomy versus radical nephrectomy in patients with small renal tumors--is there a difference in mortality and cardiovascular outcomes?. J Urol.

